# COVID‐19 and the gain of function debates: Improving biosafety measures requires a more precise definition of which experiments would raise safety concerns

**DOI:** 10.15252/embr.202153739

**Published:** 2021-09-03

**Authors:** Kelsey Lane Warmbrod, Michael G Montague, Gigi Kwik Gronvall

**Affiliations:** ^1^ Johns Hopkins Center for Health Security Baltimore MD USA; ^2^ University of Washington Seattle WA USA; ^3^ Department of Environmental Health and Engineering Johns Hopkins Bloomberg School of Public Health Baltimore MD USA

**Keywords:** Economics, Law & Politics, Microbiology, Virology & Host Pathogen Interaction, Science Policy & Publishing

## Abstract

The COVID‐19 pandemic has rekindled debates about gain‐of‐function experiments. This is an opportunity to clearly define safety risks and appropriate countermeasures.

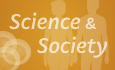

The so‐called “gain of function” research has been recently debated in the context of viral research on coronaviruses and whether it is too risky to undertake such experiments. However, the meaning of “gain of function” or “GOF” in a science policy context has changed over time. The term was originally coined to describe two controversial research projects on H5N1 avian influenza virus and was later applied to specific experiments on coronavirus. Subsequent policies and discussions have attempted to define GOF in different ways, but no single definition has been widely accepted by the community. The fuzzy and imprecise nature of the term has led to misunderstandings and has hampered discussions on how to properly assess the benefit of such experiments and biosafety measures.

The fuzzy and imprecise nature of the term GOF has led to misunderstandings and has hampered discussions on how to properly assess the benefit of such experiments and biosafety measures

## The original “Gain of Function” research

During the early 2000s, H5N1 avian flu virus infected people with high rates of mortality—exceeding 60%; but fortunately, the virus had only limited person‐to‐person transmission (CDC, 2015). There were concerns though that it might evolve to transmit more efficiently among humans while retaining its high mortality. Two laboratories independently sought to determine genetic markers associated with mammalian transmission, which could be used for public health surveillance (Herfst *et al*, 2012; Imai *et al*, 2012). In so doing, they created non‐naturally occurring viruses with higher transmissibility in mammals. When both research groups attempted to publish their findings, the US National Science Advisory Board for Biosecurity, an advisory committee to the Director of the NIH, requested that publication be halted while the security implications of publishing were examined. They were concerned that details of these experiments, including the specific genetic changes associated with transmissibility, would enable nefarious actors to create an influenza‐based biological weapon. In early 2012, an international group of influenza researchers announced a 60‐day pause on research with highly pathogenic avian H5N1 viruses that could lead to enhanced transmissibility in mammals. The 60‐day pause eventually lasted more than 8 months, during which the topic of whether or how the research should be conducted and shared was hotly debated in forums assembled by the WHO, the US National Academies, and in the pages of newspapers and scientific journals. Eventually, both research teams could proceed with publication, and the unredacted articles—including the full sequence data—were published in *Science* and *Nature* in 2012.

Both sets of H5N1 experiments included introducing mutations to the influenza genome to observe resulting changes in phenotype related to transmissibility. The research team under Yoshihiro Kawaoka introduced random mutations within a 143–amino acid stretch of the globular head of the influenza hemagglutinin surface protein. Ferrets were infected with the mutated viruses and placed in adjacent cages with uninfected ferrets so that viral particles could travel between the cages. The team found that several mutants were able to transmit between ferrets (Imai *et al*, 2012). The team under Ron Fouchier introduced mutations previously identified to be important in host range determination and receptor binding and used adjacent cages to see if their influenza mutants would transmit between ferrets, similar to the Kawaoka team. When they found no evidence of transmission, they serially passaged the mutated viruses for ten passages to allow the mutated viruses to adapt to ferrets. Following passaging, adjacent cages were again used to test for transmission, which was successful, demonstrating that H5N1 could evolve to become transmissible between mammals (Herfst *et al*, 2012).

A couple years later, an unrelated series of biosafety and biosecurity incidents prompted another examination of GOF work. In 2014, researchers at the CDC had been accidentally exposed to anthrax, highly pathogenic influenza strains were accidentally sent by the CDC to clinical laboratories, the US Department of Defense accidentally shipped incompletely irradiated live anthrax spores to dozens of diagnostic laboratories, and an abandoned box in an NIH cold room was found to contain glass vials of smallpox samples, which should have been turned over to the WHO or destroyed decades earlier. These biosafety and biosecurity failures, though completely unrelated to influenza research, led the White House Office of Science and Technology Policy to issue a funding pause on GOF studies involving influenza, MERS, and SARS. It only applied to research with these three viruses that might result in enhanced pathogenicity and/or transmissibility in mammals via the respiratory route. This funding pause was in place until the Recommended Policy Guidance for Departmental Development of Review Mechanisms for Potential Pandemic Pathogen Care and Oversight (P3CO) policy was implemented in 2017 (Department of Health & Human Services, 2017). Currently, it applies only to research conducted at or funded by the US Department of Health and Human Services, which includes the NIH.

P3CO created a review mechanism for research with pathogens “with pandemic potential,” which the policy defined as a pathogen with high transmissibility and virulence. The P3CO policy marked a shift away from the GOF language toward potential pandemic pathogen (PPP) wording to describe research of concern. In fact, PPP describes the true concern—the potential release of a pathogen that could cause widespread harm to public health—better than the more vague term GOF.

… PPP describes the true concern—the potential release of a pathogen that could cause widespread harm to public health—better than the more vague term GOF

## Benefits of GOF research

It is always challenging to appropriately measure direct benefits from basic research, including GOF research. Still, it has yielded tangible benefits. Research on highly pathogenic avian influenza has identified mutations that contribute to enhanced transmissibility in mammals and which are a warning signal when observed in environmental surveillance. In the case of H5N1 and the original GOF research, all the mutations created in the laboratory were already circulating in naturally occurring virus strains but were not encompassed in one strain. Medical countermeasure development has also benefited from GOF research, allowing researchers to determine the best targets for broad‐acting therapeutics. A 2015 analysis of risk and benefits of GOF research noted that GOF experiments help researchers estimate the speed at which escape mutants to vaccines might be generated or resistance may evolve in response to selection pressures, even if the exact mutations are not directly relevant to human hosts (Casagrande *et al*, 2015). For coronavirus research, GOF‐like experiments may also become necessary to develop broad‐based vaccines and therapeutics.

Medical countermeasure development has also benefited from GOF research, allowing researchers to determine the best targets for broad‐acting therapeutics

There is some additional imprecision for coronaviruses of the already imprecise GOF term: as SARS was already adapted to human ACE2 receptors—unlike the H5N1 avian influenza, which was not adapted to humans—Ralph Baric, a prominent CoV expert from the University of North Carolina stated that transmissibility studies for some coronaviruses would not be appropriately considered GOF (Potential Risks & Benefits of Gain‐of‐Function Research: Summary of a Workshop, 2015).

## Definitions of GOF

Defining what is GOF remains challenging and people will often disagree on what does or does not count as GOF. One example of recent disagreement is a 2015 article that reported an experiment whereby the spike protein of a SARS‐like virus circulating in bats was inserted into a SARS‐CoV‐1 backbone (Menachery *et al*, 2015). By inserting only the spike protein into the SARS‐CoV‐1 backbone, the researchers reduced other variables that could be contributing to pathogenesis to better understand the impacts of the spike protein itself. They found that the transgenic virus was able to replicate as well as SARS‐CoV‐1 and therapeutics against SARS‐CoV‐1 were not particularly effective against. During the COVID‐19 pandemic, this article gained widespread attention with some arguing that this was GOF work because a transgenic virus was created, and others saying it was not because the bat virus backbone was not significantly different from the SARS‐CoV‐1 backbone.

Whatever the terminology, such experiments help scientists understand how close a novel pathogen is from mammalian transmission. For example, scientists can look for the lowest number of mutations needed for a naturally circulating pathogen in animals to be able to infect humans. One mutation away from spillover may incentivize a robust and well‐funded intervention by public health authorities, such as implementing agricultural biosafety measures to prevent contact between different animal populations or between humans and animal populations, whereas 20 mutations may just put the pathogen on a watch list. Scientists may also explore how different environments affect evolution or the impacts of specific mutations in different environments, which can inform public health mitigation measures. For example, a mutation that has no impact on virulence in one population may cause increased virulence in another population based on host genetics or underlying comorbidities. Alternatively, certain mutations may make a pathogen more stable in one environment over another, which may inform the choice of disinfectants or hygiene measures.

Medical countermeasure development is one of the major benefits of GOF research. One of the most basic experiments is repeated passaging in animal models to create attenuated strains of the virus that are better adapted to the animal and less pathogenic for humans. This procedure has yielded many vaccines, including the yellow fever vaccine that is currently in use. This is similar to what Fouchier’s team did with H5N1 in ferrets, following site‐directed mutagenesis. Potential GOF research for MCM development can also identify therapeutics that are more resistant to escape mutants.

There is also a risk of *not* conducting GOF research because vital information may be otherwise missed. GOF research has helped build our understanding of CoVs before the pandemic and gave researchers an understanding of the basic patterns and rate of evolution and characterized spike proteins in different backbones; both have contributed to our ability to make effective vaccines quickly. Inserting mutations observed from public health genomic surveillance into other viral backbones can also help researchers test the efficacy of vaccines against emerging variants (Plante *et al*, 2021).

Opponents to GOF research believe that it poses an unjustified risk to public health. They are concerned that a modified virus could escape from a laboratory, spread person to person, and potentially spark a pandemic. They have also been concerned that some GOF research could be intentionally misused for nefarious purposes. Such experiments are usually classified as “dual‐use research of concern” or DURC, which is “life sciences research that, based on current understanding, can be reasonably anticipated to provide knowledge, information, products, or technologies that could be directly misapplied to pose a significant threat with broad potential consequences to public health and safety, agricultural crops, and other plant, animal, the environment, materiel, or national security.” DURC involving regulated pathogens is already subject to considerable oversight. Although the extent of dual‐use risks in non‐regulated pathogens may never be fully accounted for, scientists should nonetheless clearly articulate the benefits before starting any research that could be considered GOF.

## Challenges in defining GOF and governance

Initially, the primary concern with GOF research was about security: could someone steal a modified pathogen or use a publication to recreate a transmissible pathogen to deliberately cause an outbreak? Over time, the potential safety has taken precedence as the main concern of its opponents: could a modified pathogen accidently be released from a laboratory to cause an outbreak? Without a consensus definition of what GOF actually is, however, it has been challenging to define what is of concern and how to design appropriate oversight mechanisms. There are several US government documents and WHO documents about GOF or DURC, but the term remains vague. Oversight that is too broadly defined would impose regulatory burdens unnecessarily and potentially limit the ability of the research community to conduct vital research, whereas oversight that is too narrowly defined could potentially allow truly concerning research to proceed without oversight.

Over time, the potential safety has taken precedence as the main concern of its opponents: could a modified pathogen accidently be released from a laboratory to cause an outbreak?

In genetics, “gain of function” usually refers to a mutation that results in an enhanced phenotype compared with the wild‐type allele. For example, a mutation that increases metabolism of a substrate by a factor of 2 would be considered a gain of function. From a technical standpoint, nearly all microbial evolution work could therefore be considered “gain of function” because any experiment that forces a microbial population to evolve could result in a pathogen with new or enhanced characteristics, which may increase a pathogen’s fitness, virulence, or transmissibility.

Transmissibility and pathogenicity are complex traits, and they are not well‐understood. A “gain of function” mutation does not necessarily cause an increase in virulence or pathogenesis. Additionally, one mutation may affect several different traits, some of which may increase fitness, whereas others decrease fitness. A mutation may enhance fitness in one environment but not in another: even if one trait is enhanced in a laboratory, that does not automatically mean the pathogen will be more successful in a human population. Vice versa, experiments that have nothing to do with virulence or transmissibility may inadvertently create a strain with higher virulence or transmissibility, and unless these traits are endpoint measures, the researchers will not know they have created enhanced pathogens.

Evolution, especially in pathogens, is complex and not linear. This makes it difficult to determine whether specific research activities do create unjustified risks and make it difficult to predict the outcomes of artificial selection pressure on a microbial population. Researchers would not know the exact outcome until after the experiment. If the experiment is repeated, the exact same results are not guaranteed as different mutations may arise even in the same environment. This is especially true for viral populations that exist as quasispecies, that is, a large population of closely related but genetically diverse variants on which selection acts on the population as a whole, rather than a single variant. One variant in the population may have mutations that confer enhanced transmissibility or pathogenicity, but that variant is only one part of the whole. If the entire population is not fit enough to survive in a given environment, even the most transmissible of variants will not survive. Additionally, any manipulation of a pathogen, even if just passaged once, introduces selection pressures, which may change the population. Does this mean that any work with an infectious pathogen should be considered GOF? Or should GOF only refer to deliberate, directed genetic engineering? The former is too broad: even diagnostic laboratories would be considered to be conducting GOF experiments through their normal activities. The latter is too narrow: Kawaoka’s H5N1 experiments would not count as GOF because his team used random mutagenesis.

Accurately defining the problem and outlining areas of research that are of concern are critical for effective and sustainable governance. A list‐based approach creates a risk that low‐risk work is inappropriately included. For example, the US Federal Select Agent Program provides oversight for all research with any microbial agents or toxins included on the Select Agents and Toxins List (https://www.selectagents.gov/sat/list.htm). All research with these agents, regardless of the risk level, is automatically governed by the select agent rules. This is in fact helpful as it gives oversight for all research with pathogens that pose exceptionally high risk to public health. However, list‐based approaches also inherently mean that some things that may be just as risky are excluded. For instance, experiments to expand the host range of a pathogen not listed would not be covered under this governance framework. List approaches can also become outdated as technology advances.

Accurately defining the problem and outlining areas of research that are of concern is critical for effective and sustainable governance

## What functions are most concerning?

Clearly, the term “gain of function” is overly broad and applies to most microbiological research. Even something as simple as growing cells in a dish is a “passage” and provides an opportunity for mutate under the selective conditions which in turn could cause a gain of some function. However, the four functions that cause the most concern are in the context of pathogens: gain of host range, transmissibility, pathogenicity, and escaping medical countermeasures.

… the four functions that cause the most concern are in the context of pathogens: gain of host range, transmissibility, pathogenicity, and escaping medical countermeasures

Once one step away from GOF, certain commonalities and trends become apparent. Notably, most represent functions or biological traits of a pathogen on the scale of the whole pathogen infecting its host. Pathology is, for example, a complex interplay between the infection process and the host immune responses often across many different organs and tissue types. Transmission includes all of that and the complexity of social interactions at the community level. The host range adds the dynamics of inter‐species contacts between various hosts. The evasion of medical countermeasures is the only class of research that might be structured in such a way that specific drug targets or specific tissue types are studied without the context of the whole host or pathogen. This is important as such carefully structured studies would make any prospect of laboratory escape much harder, especially if the pathogen does not need to be in a complete form for the research. For example, the spike protein of SARS‐CoV‐2 in a mammalian expression vector or as part of a pseudovirus assay is not a biosafety risk.

Even if the experiments themselves are as safe as possible, there is another danger from GOF experiments: “information hazards.” Some experiments could potentially represent a danger from the publication or mere knowledge of the research itself. Nick Bostrom, a philosopher at Oxford University, has published a typography of information hazards, a full discussion of which is beyond the scope of this article (Bostrom, 2011). Oversight to mitigate information hazards should be considered and implemented separately from the regulation of laboratories.

## Balancing risk and benefits

With nearly any definition of GOF, there will be measures that can be implemented to mitigate risk, including biosafety measures to reduce the risk of accidental release. Following the H5N1 experiments, several agencies updated guidance documents to recommend that research with H5N1 should be conducted in BSL3 or BSL3+ laboratories to minimize the biosafety risks and/or recommend that laboratory staff should be vaccinated against H5N1 (Russell *et al*, 2018). There are methodological measures to lower the risk, such as using pseudoviruses—a viral particle that is unable to fully replicate on its own. However, such methodological changes may not be possible in all cases or for all research questions.

It is important that diverse stakeholders are included in the risk and benefit assessments. Scientists, medical providers, ethicists, policy makers, and the public all have a say about this research. Technical expertise, however, is critical to both understanding of the potential risks and methods to reduce them; it is the abstraction of these risks into plain language that introduced the unworkable, vague, and easily misconstrued “gain of function” language into the policy lexicon.

The people who do the research should be aware of the safety and security risks associated with gains of transmissibility, pathogenicity, and evasion of medical countermeasures, and how this relates to biocontainment should be part of the review of this research. Most life scientists do not receive training in biosecurity and would therefore benefit from better knowledge of ethics, safety, and security.

Regarding biosafety, there is an ISO standard for risk management and multiple international and national efforts for enhancing biosafety. However, there is a lack of funding to study applied biosafety and identify practices that are most effective or to explore novel solutions. More funding for this field and pushing for biosafety to become its own field of research would be helpful for risk mitigation across the life sciences. In the past, the BWC has provided a forum for stakeholders to discuss and strengthen biosafety internationally. Biosafety training has been a priority in several countries, in part to address the requirements for information sharing required by the BWC. Research institutions, funders, publishers, policy makers, clinical laboratories, educational institutions, private companies, and governmental agencies all have a stake in these discussions, and each can implement their own measures to mitigate risks even if none of these groups could address the problem in its entirety.

## Registered reports

Another partial solution to the oversight of potentially dangerous biological research might be the Registered Report. Introduced by Christopher Chambers in 2013, the Registered Report is a publishing model for empirical sciences in which a study is peer‐reviewed for concept and methodology and accepted or rejected for publication *before* the experiments are performed or the results known (Chambers, 2013). The psychological sciences first adopted this publication model as a response and partial remedy to the “reproducibility crisis,” but it has also become popular in the life sciences. Clinical trials have been using a similar model to ensure sound methodology and appropriate consideration of ethics before a trial in humans (preprint: Chambers & Tzavella, 2020). This publishing model is intended to remedy issues of publication bias, hindsight bias, and selective reporting bias as negative results get published as often as positive ones. Registered Reports also prevent studies from being designed and carried out with inadequate statistical power or other flawed methodologies as peer review identifies such issues prior to acceptance.

In this way, Registered Reports are a *de facto* scientific self‐oversight mechanism, one that might be used for biosafety self‐oversight just as effectively as for statistical and scientific rigor. Unlike classical publishing models, which review results and experiments only after the fact, a Registered Reports provides peer review of the study design before it is performed and thus in time to recommend and implement additional safety steps. Unlike institutional review boards, which are a common biosafety review step that also happens before potentially dangerous research is conducted, Registered Reports intentionally selects academic peers in the same or closely related disciplines and from outside the institution conducting the research, which allows input from other experts and removes the potential for institutional conflicts of interest. This could supplement, rather than replace, IRBs, and one could envision that an IRB would require a Registered Reports publication mode for potentially dangerous research. Just as Registered Reports in the psychological sciences removes result‐dependent biases on reporting, it would improve reporting of biosafety practices and failures.

… the debate should shift from where it has been to a more productive, pragmatic, and technical discussion of actual risks and means to mitigate them

As the COVID‐19 pandemic continues, the debate over GOF research is likely to continue. However, the debate should shift from where it has been to a more productive, pragmatic, and technical discussion of actual risks and means to mitigate them. Identifying what is actually of concern should be the first step, followed by a focus on policies and interventions. There are options for reducing risk—improved biosafety measures, alternative technical methodologies, and registered reports, as well as other potential solutions—but without a common understanding of the problems and good‐faith efforts to address them, political debates will continue to provide heat and not light.

## Conflict of interest

The authors declare that they have no conflicts of interest.
